# Refractory ascites—the contemporary view on pathogenesis and therapy

**DOI:** 10.7717/peerj.7855

**Published:** 2019-10-15

**Authors:** Beata Kasztelan-Szczerbinska, Halina Cichoz-Lach

**Affiliations:** Department of Gastroenterology with Endoscopy Unit, Medical University of Lublin, Poland

**Keywords:** Refractory ascites, Liver crirrhosis, Diuretics, Paracentesis, Treatment

## Abstract

Refractory ascites (RA) refers to ascites that cannot be mobilized or that has an early recurrence that cannot be prevented by medical therapy. Every year, 5–10% of patients with liver cirrhosis and with an accumulation of fluid in the peritoneal cavity develop RA while undergoing standard treatment (low sodium diet and diuretic dose up to 400 mg/day of spironolactone and 160 mg/day of furosemide). Liver cirrhosis accounts for marked alterations in the splanchnic and systemic hemodynamics, causing hypovolemia and arterial hypotension. The consequent activation of renin-angiotensin and sympathetic systems and increased renal sodium re-absorption occurs during the course of the disease. Cirrhotic patients with RA have poor prognoses and are at risk of developing serious complications. Different treatment options are available, but only liver transplantation may improve the survival of such patients.

## Introduction

Liver cirrhosis and its complications are significant problems in Poland, as well as in populations of Western Europe and North America. According to National Vital Statistics Reports published in 2018, liver cirrhosis ranks 12th among the most common causes of death in the USA (Heron, 2018). The accumulation of ascitic fluid in the peritoneal cavity, a sign of decompensation, occurs in about 60% of patients within 10 years of the disease course. The appearance of ascites in the course of cirrhosis indicates an unfavorable prognosis. Statistical data of 35 observations show that mortality in this group of patients may reach 40% within 1 year and 50% within 2 years (Senousy & Draganov, 2009).

Ascites refractory to treatment is one of the most serious complications caused by decompensated liver cirrhosis. Resistance to conventional therapy develops in 5–10% of patients with cirrhotic ascites within a year of treatment (Siqueira, Kelly & Saab, 2009; Salerno et al., 2010). When an insufficient natriuretic effect is observed, or more often, complications from treatment, the withdrawal of diuretics is recommended. From the moment of RA diagnosis, the average survival period of patients decreases to approximately 6 months (Siqueira, Kelly & Saab, 2009).

## Survey methodology

A Medline search was performed based on key words that included the following terms: refractory ascites (RA), liver cirrhosis and treatment. Only reports published in English and human studies were included. The search covered 377 papers published between 2005 and 2018.

## Definition of ra

According to the International Ascites Club criteria (IAC), the term “refractory ascites” refers to ascitic fluid that cannot be mobilized or that has an early reoccurrence (e.g., after paracentesis) that cannot be prevented by treatment (Senousy & Draganov, 2009; Siqueira, Kelly & Saab, 2009; European Association for the Study of the Liver, 2010; Salerno et al., 2010). It is vital to remember that the evaluation of patient response to diuretics and to a reduction of dietary sodium should be performed in clinically stable patients without any additional complications, such as bleeding or infection. In 1996, the IAC recommended the classification of RA into two subtypes: (1) diuretic-resistant ascites—when a patient does not respond to the maximum dose of diuretics and (2) diuretic-intractable ascites—for a patient presenting with complications of diuretic therapy that preclude using an effective dose of diuretics (European Association for the Study of the Liver, 2010).

In 2003, the diagnostic criteria for RA have been revised and they are as follows (Moore et al., 2003; Cardenas & Arroyo, 2005; European Association for the Study of the Liver, 2010):

1. Treatment duration: patients must be on intensive diuretic therapy (spironolactone 400 mg/day and furosemide 160 mg/day) for at least 1 week and on a salt-restricted diet of fewer than 90 mmol or 5.2 g of salt/day.
2. Lack of response: mean weight loss of 0.8 kg over 4 days and urinary sodium output less than the sodium intake.
3. Early ascites recurrence: the reappearance of grade 2 or 3 ascites within 4 weeks of initial fluid mobilization, when minimal or no ascites is achieved.
4. Diuretic-induced complications: diuretic-induced hepatic encephalopathy (HE) is the development of encephalopathy in the absence of any other precipitating factor. Diuretic-induced renal impairment is a >100% increase of serum creatinine to a value of >2 mg/dL in patients with ascites responding to treatment. Diuretic-induced hyponatremia is defined as a decrease of serum sodium by >10 mmol/L to a serum sodium level of <125 mmol/L. Diuretic-induced hypo- or hyperkalemia is defined as a change in serum potassium level to <3 or >6 mmol/L, despite appropriate measures (Moore et al., 2003; Cardenas & Arroyo, 2005; European Association for the Study of the Liver, 2010).

Randomized trials indicate that approximately 5–10% of patients with cirrhosis and ascites become refractory to standard therapy (Siqueira, Kelly & Saab, 2009; Salerno et al., 2010). Refractory ascites frequently coexists with type 2 hepatorenal syndrome, spontaneous bacterial peritonitis (SBP), hyponatremia, muscular dystrophy and/or pleural effusion. When the diagnosis of RA is established, a prompt commencement of intensive therapeutic measures and patient referral to a liver transplant center is recommended.

## The pathogenesis of ascites in liver cirrhosis

Currently, there are three hypotheses, i.e., the underfilling theory, overflow theory and peripheral arterial vasodilation theory, to explain the reason for forming ascites in end-stage liver disease (Fukui, 2015). The formation of ascites in patients with cirrhosis is influenced by two factors: portal hypertension (PH) and renal sodium retention (Cardenas & Arroyo, 2005; Kashani et al., 2008; Salerno et al., 2010). Portal hypertension contributes to increased resistance to blood flow at the level of hepatic sinusoids and leads to the development of hepatic sinusoidal PH. Consequently, a backward transmission of the increased pressure reaching the visceral capillaries leads to distention and the penetration of the fluid into the peritoneal cavity. The increased sinusoidal pressure causes peripheral, predominantly visceral and arterial, vasodilatation acting through locally released vasoactive factors, mainly nitric oxide, but also glucagon, prostacyclin, vasoactive intestinal peptide, substance P and platelet-activating factor (Kashani et al., 2008; Senousy & Draganov, 2009). Visceral vasodilation increases blood volume in the visceral area and further enhances portal pressure, but also leads to the reduction of the systemic blood volume. Furthermore, systemic hypovolemia stimulates the neurohormonal mechanisms responsible for sodium retention, which are intended to counterbalance the decreased blood volume and to fill in the expanded vascular bed. Activation of the renin-angiotensin-aldosterone axis (RAA), adrenergic nervous system and antidiuretic hormone (vasopressin) plays a relevant role in this process (Senousy & Draganov, 2009; Siqueira, Kelly & Saab, 2009; Salerno et al., 2010). At the same time, there is a gradual decline in both kidney perfusion and glomerular filtration. Sodium reabsorption increases significantly in the proximal section of the nephron loop, and its delivery to the distal segments of the nephron consequently decreases. Thus, sodium renal retention appears proximally to the site of action of aldosterone antagonists and loop diuretics (Salerno et al., 2010). This explains the lack of effective diuretic treatment in some cirrhotic patients. Additionally, the reduced cardiovascular response to vasoconstrictive factors support the state of a relative deficiency of arterial blood volume and augment the hypovolemic effect of diuretics. Such circumstances reveal side effects of the aforementioned medications and make treatment impossible to continue. Thus, resistance to diuretics may be a consequence of hemodynamic disturbances arising in the course of advanced liver cirrhosis (Cardenas & Arroyo, 2005). As a result of both hemodynamic and renal disorders, there is progressive fluid penetration from the hepatic sinusoids and visceral vessels, and its accumulation inside the peritoneal cavity. As liver failure progresses, the degree of sodium retention (determined by the amount of sodium excreted in the urine) and hyponatremia correlate with the survival rate of cirrhotic patients. The pathogenesis of hepatorenal syndrome resembles the pathogenesis of ascites. It is believed that RA is a pre-hepato-renal syndrome and is, in fact, a common clinical manifestation of type 2 hepatorenal syndrome (Cardenas & Arroyo, 2005; Salerno et al., 2010).

## So-called false-refractory ascites

A lack of or inadequate response to diuretics is sometimes observed in certain clinical situations that cannot be labeled as RA (Senousy & Draganov, 2009; European Association for the Study of the Liver, 2010; Salerno et al., 2010). Therefore, the correctness of therapy should be assessed first. Loop diuretics (which worsen hyperaldosteronism) as monotherapy or insufficient doses of aldosterone antagonists (relative to the degree of RAA axis activation) are not the recommended therapies. In such situations, the response to treatment can be restored by adjusting the doses. Similarly, unnecessary high doses of diuretics induce excessive diuresis leading to a negative fluid balance, inadequate weight reduction and pre-renal kidney injury. Temporary resistance of ascites to treatment may occur in the case of impaired renal function due to an iatrogenic or concomitant, but transient, disturbance of patient’s health status.

The iatrogenic refractoriness of ascites can be caused by medications such as non-steroidal anti-inflammatory drugs that interfere with renal function by decreasing prostaglandin synthesis; ACE inhibitors, which act as vasodilatators; and angiotensin receptor blockers, which reduce renal perfusion and glomerular filtration rate. Comparable side effects may be observed during nephrotoxic treatment, e.g., aminoglycoside administration (European Association for the Study of the Liver, 2010).

Disorders manifesting with fluid loss due to vomiting, diarrhea and bleeding may also promote kidney dysfunction and an altered response to diuretics.

Infections like spontaneous bacterial peritonitis enhance vasodilatation and promote an imbalance between intravascular blood volume and vascular bed capacity. In such clinical cases, discontinuation of the harmful medication or removal of the factor causing changes in the intravascular fluid volume may restore the appropriate response to the standard ascites treatment. Furthermore, one should also remember the supposed resistance of ascites in the case of a non-compliant patient who does not strictly follow a low-sodium diet (≤90 mmol/day). Verification of this clinical setting is possible on the basis of the calculation of daily sodium urine excretion (a daily sodium balance), as well as the analysis of fluctuations in the patient’s body weight over the last weeks (an increase in patient’s body weight) (Cardenas & Arroyo, 2005).

## Approach to a patient with refractory ascites

Before making the right therapeutic decision, one should confirm the diagnosis of RA and rule out other causes of resistance to treatment. Such an approach is necessitated by the fact that approximately 5% of patients with ascites have more than one cause of fluid accumulation in the peritoneal cavity, e.g., the patient may have liver cirrhosis and tumor dissemination in the peritoneal cavity, which significantly changes the response to diuretic therapy and may give rise to the incorrect interpretation of ascites resistance to treatment (Senousy & Draganov, 2009).

The serum ascites albumin gradient (SAAG) is a helpful tool for the pathophysiological classification of ascites into two types: with a high gradient (SAAG ≥ 1.1 g/dL) indicative of PH (97% sensitivity) (Runyon et al., 1992), or with a low gradient (SAAG < 1.1 g/L) unrelated to PH. For the best accuracy of the formula, the two parameters (i.e., serum albumin and ascitic albumin levels) should be measured at the same time. Furthermore, in cases with SAAG ≥ 1.1 g/dL, determination of an ascitic fluid total protein level helps to distinguish cardiogenic and cirrhosis related causes of ascites. The protein concentration greater than or equal to 2.5 g/dL points at cardiac causes of ascites (Caldwell & Battle, 1999; McGibbon et al., 2007).

Doppler ultrasonography and serum alpha-fetoprotein levels are useful tools for the detection of portal vein thrombosis or hepatocellular carcinoma, respectively. In these scenarios, the lack of response to diuretic therapy occurs due to the disease features.

The ideal method for ascites treatment is still unavailable. It should ensure efficient fluid mobilization from the peritoneal cavity, prevent its recurrence, improve patient’s comfort and survival and directly affect the mechanism of ascites formation instead of being only a method of mechanical fluid evacuation from the abdominal cavity.

## Diuretics

In the majority of patients with RA, diuretic therapy has no effect in preventing or delaying ascites recurrence after paracentesis. Diuretics should be completely discontinued if complications (i.e., HE, impaired renal function, electrolyte disturbances) occur. Remaining patients should continue the treatment only when the excretion of sodium in the urine is greater than 30 mmol/day (European Association for the Study of the Liver, 2010).

Despite the lack of response to diuretic therapy, it is still very important for patients to follow a low sodium diet and to stay educated in this regard (such a diet has an effect on the rate of ascitic fluid accumulation) (Senousy & Draganov, 2009). Daily fluid restriction is indicated only in cirrhotics with ascites, whose serum sodium level is less than 130 mEq/L (Runyon & AASLD Practice Guidelines Committee, 2009; Senousy & Draganov, 2009; European Association for the Study of the Liver, 2010).

Currently, several methods of RA treatment can be implemented, but none are entirely acceptable (Runyon & AASLD Practice Guidelines Committee, 2009; European Association for the Study of the Liver, 2010):

1. Large-volume paracentesis (LVP) and intravenous albumin supplementation;
2. Transjugular, intrahepatic portosystemic shunt (TIPS);
3. Automatic, low-flow pump for ascitic evacuation (ALFApump System);
4. Cell-free and concentrated ascites reinfusion therapy (CART);
5. Liver transplantation;
6. Vasopressors, that improve patient sensitivity to diuretics.

## Large-volume paracentesis

Large-volume paracentesis with intravenous albumin infusion (six to eight grams for each liter of ascitic fluid dropped) remains the standard treatment of RA. Albumin infusion is not required when the volume of fluid evacuated is less than four to five liters (Senousy & Draganov, 2009; European Association for the Study of the Liver, 2010; Salerno et al., 2010). Paracentesis is considered a safe procedure with a low risk of serious complications, even in patients with coagulopathy (De Gottardi et al., 2009). Runyon estimates the risk of paracentesis-related abdominal wall hematoma as 1%, and the risk of bleeding into the peritoneal cavity or iatrogenic infection to be approximately 1 in 1,000 (Runyon & AASLD Practice Guidelines Committee, 2009). There is no significant benefit of transfusing fresh frozen plasma (FFP) or platelets to prevent bleeding from paracentesis. Fresh frozen plasma may be administered depending on the indications in individual cases, but it is not the standard treatment for every paracentesis case (Biecker, 2011). The INR value, above which paracentesis should not be performed, is not clearly defined. Pache & Bilodeau (2005) analyzed over 4,500 cases of paracentesis in their retrospective study and confirmed the good tolerance of this procedure, even in patients with INR up to 8.7 and platelet numbers as low as 19,000/μL.

However, a common complication of the procedure is the leakage of fluid from the abdominal wall puncture site. The complication can be avoided by using a special technique known as the Z-track technique (Runyon & AASLD Practice Guidelines Committee, 2009; Senousy & Draganov, 2009; Salerno et al., 2010), where, prior to needle insertion, one pulls the skin about two centimeters in the caudal direction and then performs a puncture in the abdominal wall.

After paracentesis is completed and the needle is removed, the skin returns to its original position, and the external opening on the skin does not communicate in a straight line with the internal orifice in the peritoneal cavity, which prevents leakage. Another way to prevent leakage is to place a patient on a flank opposite to the site where the puncture is made for about 2 hours. If there is an ascitic fluid leak, which cannot be inhibited by the aforementioned methods, a surgical suture should be applied at the puncture site. Many clinicians recommend performing LVP instead of multiple dropping of smaller (four to six L) amounts of fluid (Senousy & Draganov, 2009; Siqueira, Kelly & Saab, 2009). Arguments for such a proceeding are a quicker comfort improvement, reduction in the risk of complications associated with multiple needle insertion into the peritoneal cavity, and lower risk of fluid leakage after paracentesis. However, the most serious complication after LVP seems to be circulatory disorders (Senousy & Draganov, 2009; Nasr et al., 2010; Salerno et al., 2010). They appear approximately 12 hours after the performing paracentesis and are manifested by an increase in plasma renin activation and stimulation of the sympathetic nervous system to values greater than those observed before the procedure.

Paracentesis-induced circulatory dysfunction (PICD) is defined as an increase in plasma renin activity by more than 50% of the original value, to a value of more than 4 ng/mL/h on day 6 after paracentesis (Senousy & Draganov, 2009; Salerno et al., 2010). Although in the majority of cases this is a clinically asymptomatic or mild condition, it has a negative effect on the course of the disease by increasing the incidence of hyponatremia and renal disorders, and its severity is inversely correlated to patient survival. The most common adverse effects after removal of more than five liters of ascetic fluid include weakness, dizziness and syncope. Intravenous albumin supplementation prevents these adverse consequences of paracentesis. It reduces the incidence of PICD to 15–20% (Moreau et al., 2006; Senousy & Draganov, 2009; Nasr et al., 2010). Twenty percent intravenous albumin solution is available in Europe. It was found that other preparations that increase the volume of human plasma, such as dextran, hydroxyethylated starch or saline, do not have an equally beneficial effect on the prophylaxis of circulatory disorders induced by paracentesis (Salerno et al., 2010). The half-life lengths of the preparations, which in the case of albumin is the longest (21 days), are probably significant. Moreover, albumin effectively prevented hyponatremia in comparison with other colloids (8% of 482 patients vs 17% of 344 patients) (Salerno et al., 2010), and the number of liver complications observed was also significantly lower in that group of patients (Moreau et al., 2006).

It should be emphasized that patients with liver cirrhosis should not receive hydroxyethylated starch after paracentesis. It has been shown that it is absorbed by Kupffer cells and stored in their lysosomes. As a consequence, an enhancement of portal pressure may occur and the risk of bleeding from esophageal varices increases (Runyon & AASLD Practice Guidelines Committee, 2009).

Sersté et al. (2011) published the results of studies investigating the impact of beta-blockers on the risk of paracentesis-induced circulatory disorders. Reports suggest that beta-blocker treatment may increase the incidence of PICD in patients with liver cirrhosis and RA. If the aforementioned data are confirmed, the prophylaxis of bleeding from esophageal varices should be modified in this group of patients.

Paracentesis provides a possibility of rapid intervention in patients with tense and massive ascites. Reducing the hepatic-venous gradient can decrease the pressure inside esophageal varices and, thus, the risk of bleeding. It has been demonstrated that paracentesis, in comparison to diuretic therapy, reduces the time of hospitalization and the incidence of complications. However, the rate of ascites recurrence and patient survival were not different in both groups (Senousy & Draganov, 2009; Siqueira, Kelly & Saab, 2009; Salerno et al., 2010).

The time interval between consecutive procedures of paracentesis may be different and probably depends on individual variations in the rate of fluid permeation, patient adherence to a low-sodium diet, distinct body structure and tolerance of abdominal fluid volume. According to recommendations from the European Association for the Study of the Liver (2010), each paracentesis should be accompanied by ascitic fluid examination (white blood cell count and smear analysis) to exclude SBP. The examination should be carried out even when the patient is asymptomatic because cases of SBP have also been reported in such patients (Romney et al., 2005; Kasztelan-Szczerbinska et al., 2011). Moreover, when there are overt signs of SBP, fluid culture and antibiogram determination are also required.

Contraindications for paracentesis: There are no absolute contraindications to the performance of paracentesis (Siqueira, Kelly & Saab, 2009; Salerno et al., 2010). However, this procedure should be avoided in patients with disseminated intravascular coagulation syndrome. Also, special attention should be paid to patients with intra-abdominal adhesions and distended urinary bladders. Ultrasound guidance helps to reduce the risk of iatrogenic complications in the above cases.

## Transjugular intrahepatic portosystemic shunt

Transjugular intrahepatic portosystemic shunt is a tract created within the liver using X-ray guidance (Garcia-Tsao, 2005; Rossle & Gerbes, 2010). This minimally invasive procedure is performed by an interventional radiologist under local anesthesia. A catheter is introduced to the hepatic vein through the jugular vein, and then to the main branch of the portal vein. The stent is placed across the hepatic vein and the portal vein and subsequently expanded by an inflatable balloon (angioplasty) to form a shunt that bypasses the liver. This artificial channel establishes a new communication route between the inflow portal vein and the outflow hepatic vein. The stent consequently reduces blood pressure within the portal vein and decompresses portal circulation. Initially, uncovered metal stents were used for the creation of TIPS. However, they were linked to frequent technical complications (i.e., shunt obstruction). Recently, polytetrafluoroethylene (ePTFE)-covered nitinol stent-grafts have been introduced and currently, they are commercially available (GORE^®^ VIATORR^®^ TIPS Endoprosthesis). Their high patency rates and survival benefits have been proven in several clinical trials (Vignali et al., 2005).

Portal hypertension causes the pressure gradient between the portal vein and the inferior vena cava (IVC) called the portal pressure gradient (PPG). The normal PPG values range from 1 to 5 mm Hg (Berzigotti et al., 2013). Direct measurements of portal pressure are highly invasive, therefore rarely used and limited to selected cases of presinusoidal PH. Currently, a hepatic venous pressure gradient (HVPG) assessment, which is the gradient between the portal vein and the hepatic vein determined as the difference between the free hepatic venous pressure and the wedged hepatic venous pressure at hepatic vein catheterization, represents the gold standard method for estimation of PPG (Thalheimer et al., 2005; Berzigotti et al., 2013). The presence of PH is confirmed when the HVPG exceeds 5 mm Hg, but only HVPG values above 10 mm Hg are associated with the risk of developing PH complications (Berzigotti et al., 2013; Abraldes, Sarlieve & Tandon, 2014). Therefore, by lowering the HVPG below 12 mm Hg, TIPS leads to the gradual disappearance of ascites. Furthermore, maintaining such pressure prevents the accumulation of ascitic fluid.

The second mechanism through which TIPS modifies PH is blood transfer from the expanded visceral circulation toward the systemic circulation and the equalization of the so-called under-filling of the vessels. As a result, there is a decrease in plasma renin activity and improvement of urinary sodium excretion (Senousy & Draganov, 2009).

The results of the conducted studies reveal that TIPS is useful for ascites control in 27–92% of patients and may induce complete resorption in about 75% of cases 1–3 months after stent insertion (Garcia-Tsao, 2005; Rossle & Gerbes, 2010; Senousy & Draganov, 2009). It should be emphasized that 95% of patients with TIPS still require diuretic therapy. Apart from its beneficial effect on the mechanism of ascites formation, TIPS improves kidney function: there is an increase in excreted urine volume and urine sodium level, as well as a decrease of serum creatinine level, and also improves the nutritional status of patients (Senousy & Draganov, 2009; Rossle & Gerbes, 2010).

Despite numerous advantages of TIPS, its insertion may be associated with several complications. They are as follows:

1. *Technical complications:* puncture of the liver capsule (approximately 33%), bleeding into the peritoneal cavity (1–2%), hemolysis and sepsis, acute renal failure (due to administration of contrast agents), cardiac arrhythmia in case of the catheter translocation into the right atrium and/or the ventricle;
2. *Hepatic encephalopathy (HE):* observed in about 30% of patients after TIPS creation, its clinical symptoms appear 2–3 weeks after the procedure; factors contributing to the HE development include: older age, advanced liver disease and previous episodes of HE;
3. *Stenosis of a stent:* the problem appears in 22–50% of patients so the patency of a stent should be monitored by Duplex Doppler ultrasonography every 3 months and by venography once a year;
4. *Intravascular hemolysis:* occurs in about 10% of patients, and its cause seems to be the direct, mechanical contact of red blood cells with a metal stent;
5. *Portosystemic myelopathy:* rare pathology, spastic muscle paralysis without coexisting sensory disorders occurring in interrelation to TIPS insertion;
6. *Decompensation of cardiac function:* the pre-load of the heart increases after TIPS insertion, which can lead to heart failure in patients with a previous history of cardiac disease; echocardiography helps to exclude patients with the left ventricular ejection fraction (LVEF) below 60% (Senousy & Draganov, 2009; European Association for the Study of the Liver, 2010; Rossle & Gerbes, 2010; Salerno et al., 2010).
7. *Portopulmonary hypertension (POPH):* develops in up to 6% of patients as a consequence of arterial vasoconstriction and remodeling of the lung vascularity induced by PH when there is a pressure gradient of >10 mm Hg, between the portal vein and the IVC called PPG. The presence of POPH should be suspected upon initial screening with transthoracic echocardiography (TTE) (Krowka et al., 2006; Fussner & Krowka, 2016). Then, a right-heart catheterization is needed for the POPH definite diagnosis considering hyperdynamic circulation and fluid overload as additional contributors to increased pressure inside the pulmonary artery in liver cirrhosis. The hemodynamic criteria for POPH include: (1) an increased mean pulmonary artery pressure (MPAP) of >25 mm Hg, (2) increased pulmonary vascular resistance of >240 dyn×s/cm^5^ and (3) pulmonary capillary wedged pressure of <15 mm Hg in the presence of PH (Benjaminov et al., 2003; Safadar, Bartolomae & Sussman, 2012). Since TIPS can temporarily increase the MPAP, contraindications to its placement include the right ventricular systolic pressure 50 mm Hg or greater, as well as an enlargement or ventricular dysfunction of the right heart on TTE (Golbin & Krowka, 2007; Fussner & Krowka, 2016). In contrast, patients with the hepato-pulmonary syndrome (HPS), the other lung complication of liver cirrhosis that presents with hypoxemia and dyspnoea secondary to intrapulmonary shunting, have been shown to benefit from TIPS procedure. The successful resolution of HPS following TIPS placement has been documented by several case reports (Wallace et al., 2012; Tsauo et al., 2015). Nevertheless, it is not recommended as a standard treatment yet and further exploration is needed in order to firmly determine the safety of this therapeutic option in cirrhotics with HPS.

There is no fully convincing evidence of TIPS impact on patient survival. The results of studies are controversial—some suggest no impact, while others suggest shortened (European Association for the Study of the Liver, 2010) or prolonged (Bai et al., 2014; Gaba et al., 2015; Bureau et al., 2017b; Rossle & Gerbes, 2010) survival after TIPS insertion. Several trials have revealed that the survival advantage weakens in 2 years after TIPS placement due to its deteriorating impact on heart function. The procedure results in systemic hemodynamic changes and may lead to cardiac overload with the development of pulmonary hypertension. Therefore, TIPS is currently primarily described as a bridging therapy in RA treatment prior to liver transplantation. Additionally, the 1-year mortality rate after TIPS implantation was significantly lower in patients treated for RA in comparison to those with variceal bleeding (Strunk & Marinova, 2018).

To augment the procedure efficacy and survival advantage, rigorous and accurate patient selection criteria play a critical role. The best candidates for TIPS placement should present with:

1. Prompt reversion of ascites and a requirement of more than three paracenteses a month;
2. Preserved liver function (i.e., bilirubin <5 mg/dL, INR <2; serum sodium level >130 mEq/L; Child-Pugh score <12; MELD score <18);
3. Age below 70 years;
4. No additional complications such as HE; progressive renal failure, infections, hepatocellular carcinoma, severe pulmonary and heart diseases (European Association for the Study of the Liver, 2010).

Data from the literature indicate that paracentesis with intravenous albumin infusion should be the first choice therapy in the treatment of RA, and TIPS placement may be considered a second-line treatment (Senousy & Draganov, 2009; Siqueira, Kelly & Saab, 2009; European Association for the Study of the Liver, 2010; Salerno et al., 2010; Burgos & Thornburg, 2018). Each patient diagnosed with RA should be urgently referred to a transplant center due to their poor prognosis (12-month survival is less than 50% likely) (Cardenas & Arroyo, 2005; Siqueira, Kelly & Saab, 2009).

## Automated low-flow ascites pump

The automated low-flow ascites pump (ALFApump) was introduced in 2011 as a new therapeutic tool for patients with RA (Stirnimann et al., 2017; Solbach et al., 2018). The device, implanted subcutaneously, drains ascitic fluid from the peritoneal cavity to the urinary bladder and facilitates spontaneous liquid elimination through urination. The fluid volume that is removed daily ranges from 500 mL to 2.5 L. The ALFApump drains small volumes of ascitic fluid in cycles every 5–10 min, making the administration of albumin not obligatory (Stirnimann et al., 2017). Since slow removal of small amounts of ascitic fluid does not significantly affect the central circulatory volume, the neurohumoral compensatory response is not aggravated. The ALFApump possesses inner sensors of both bladder and peritoneal cavity pressure, and it turns off in the case of a lack of fluid in the peritoneal cavity or the bladder being filled to its maximum capacity. The only disadvantage of ALFApump is the battery operating system which requires frequent charging (twice a day for about 20 min) (Stirnimann et al., 2017). Nevertheless, in comparison with repeat paracentesis, the effectiveness of this device, as well as the health-related quality of life it provides, is better for RA patients (Stepanova et al., 2018).

The ALFApump does not adjust the causative mechanisms of ascites formation. Currently, it is still not evident whether the pump has a significant impact on the survival of RA patients. Although, the device is effective in most patients and reduces ascites (Bureau et al., 2017a), no differences in patient survival in comparison with LVP have been confirmed so far (Fortune & Cardenas, 2017). This device is mainly used in patients with contraindication for TIPS placement or liver transplantation. Data are limited to small clinical trials. A recent study by Solbach et al. (2018) revealed a high rate of complications related to the ALFApump, such as dislocation and/or blockage of the catheter, infection and pump dysfunction, they were observed in 15 out of 21 patients (71.4%). Moreover, 21 surgical interventions were needed in 15 patients (71.4%, one to three interventions per patient). These findings may suggest that the selection of patients and surgical techniques are crucial for patient safety. Therefore, further research on this technology is required.

## Cell-free and concentrated ascites reinfusion therapy

This novel cell-free and concentrated ascites reinfusion therapy (CART) has been introduced in Japan as a modification of LVP for patients with tense ascites due to liver cirrhosis. CART was approved by the National Health Insurance in Japan in 1981 and since then, has been used in clinical settings (Hanafusa et al., 2017). It is used in the treatment of cirrhotics in patients with RA who present with diuretic resistance or diuretic intolerance that precludes their administration in higher doses. During the procedure, the filtration and concentration of ascitic fluid are followed by collected protein intravenous reinfusion (Kawaratani, Fukui & Yoshiji, 2017; Fukui et al., 2018). CART safety and efficacy in maintaining albumin concentrations were confirmed in a multicenter observational study by the Kansai CART Study Group (Takamatsu et al., 2003). Currently, the procedure is also widely used for the management of malignant ascites (Japanese Cart Study Group et al., 2011). However, the high cost of CART apparatus limits its worldwide use (Fukui et al., 2018).

## Liver transplantation

Refractory ascites impairs the quality of patient life and is a poor prognostic indicator. Less than 50% of patients with RA survive 1 year (Cardenas & Arroyo, 2005; Kashani et al., 2008; Runyon & AASLD Practice Guidelines Committee, 2009; Siqueira, Kelly & Saab, 2009). Survival rates after liver transplantation are much better (European Association for the Study of the Liver, 2010). Therefore, as a rule, once ascites becomes refractory to diuretics, liver transplantation remains the best, ultimate and the only curative treatment (Sussman & Boyer, 2011). After liver transplantation, PH completely returns to a regular state, but the reabsorption of ascitic fluid may take 3–6 months. This is probably related to persistent systemic vasodilatation and hyperkinetic circulation, which last for several months after the procedure (European Association for the Study of the Liver, 2010; Sussman & Boyer, 2011). Nevertheless, organ deficits and patient age and/or comorbidities frequently preclude the possibility to benefit from liver transplantation. Accordingly, alternative therapeutic options for RA are urgently awaited.

## Vasoconstrictive medications for the treatment of ra

During recent decades, new medical treatments using vasoconstrictive agents or selective vasopressin V2 receptor antagonists (also known as vaptans) have been introduced for treating RA (Kashani et al., 2008; Karwa & Woodis, 2009; Fukui, 2015; Zhao et al., 2018). Vasopressin plays an important role in water and sodium homeostasis. V2 receptor antagonists block the effect of the hormone on renal collecting ducts and cause water diuresis. Impairment of free water excretion and dilutional hyponatremia are the final effects of liver failure and PH, as well as are the main contributors to RA development in the course of liver cirrhosis (Arroyo et al., 1994). Combined with conventional therapy, vaptans increase the excretion of electrolyte-free water together with serum sodium concentration. Yan et al. (2015), in their meta-analysis of 14 studies containing 16 randomized controlled trials and 2,620 patients, found that vaptans could play an effective and safe role in the symptomatic treatment for RA patients who presented with an insufficient response to conventional diuretics, although no survival benefit was detected from the selected studies. Recently Kogiso et al. (2018) investigated the outcome of long-term treatment with tolvaptan. They found that it increased serum levels of albumin, decreased ammonia levels and preserved renal function after 1 year of treatment. They also concluded that a reduction in body weight after 1 week was associated with a favorable outcome of tolvaptan therapy. Common side effects of vaptans manifest with excessive serum sodium levels (>145 mmol/L) and may lead to osmotic demyelination and myelinolysis. Therefore, it is important to keep in mind that blood sodium concentration should be carefully monitored during this treatment. Furthermore, the US Food and Drug Administration (FDA) issued a warning for tolvaptan due to its hepatic toxicity leading to liver transplant or even death (Fukui, 2015). Several of vasopressin receptor antagonists have been investigated in patients with advanced liver disease (Gaglio, Marfo & Chiodo, 2012). However, none of them have gained acceptance from the FDA for the treatment of ascites in liver cirrhosis so far. Additionally, American Association for the Study of Liver Diseases (AASLD) and European Association for the Study of the Liver (EASL) guidelines do not recommend vaptans in the treatment of cirrhotic patients in light of the scarce medical evidence for their approval (Runyon & AASLD Practice Guidelines Committee, 2009; European Association for the Study of the Liver, 2018).

Vasopressors such as midodrine (α1-adrenergic agonist) (Jeffers, 2010; Misra et al., 2010; Solà & Gines, 2010; Sourianarayanane, Barnes & McCullough, 2011; Werling & Chałas, 2011) and terlipressin (the synthetic analog of vasopressin) (Krag et al., 2007; Fimiani et al., 2011) have been tested in small groups of patients with RA. They increase the effective arterial blood volume and, consequently, renal and cardiovascular function is improved in both patient groups, with and without RA.

As the physiological activity of terlipressin (vasopressin V1 receptor agonist) has been clarified, its role in RA management is being eagerly considered (Papaluca & Gow, 2018). Terlipressin has been reported to improve renal function and induce natriuresis in patients with liver cirrhosis and ascites, including those with RA (Krag et al., 2007). The synergistic effect of terlipressin and combined therapy (albumin plus diuretics) in RA patients has been recently confirmed in a prospective study (Fimiani et al., 2011). Furthermore, Gow et al. (2016) performed a small single-center pilot study to evaluate the effects of outpatient terlipressin infusion for the treatment of RA. Only five patients with the Child-Pugh C class and a mean MELD score of 18 were included in the study. A significant reduction in ascitic fluid volume removed over 4 weeks of treatment (i.e., 22.9 vs 11.9 L, *p* < 0.05) was observed. Two patients required no further paracentesis while on terlipressin infusion. Also, a significant increase in 24-h urinary sodium excretion was detected during the treatment period. The administration of terlipressin as a continuous infusion in the outpatient setting seems to be a tempting treatment option, but further trials are needed to confirm its safety and efficacy.

Midodrine that acts as a splanchnic vasoconstrictor improves renal perfusion and glomerular filtration. It is recommended by the AASLD for RA treatment (Runyon & AASLD, 2013). Midodrine combined with diuretics increases patient blood pressure and restores the sensitivity to diuretics (Fukui et al., 2018). Guo et al. (2016), in their systematic review and meta-analysis of 10 randomized controlled trials using midodrine for the treatment of cirrhotic ascites, reported that midodrine improved response rates and reduced plasma renin activity, but did not improve survival rate. Another recent report by Hanafy & Hassaneen (2016) revealed that adding rifaximin and midodrine to diuretics enhanced diuresis, improved systemic and renal hemodynamics and improved the short-term survival in patients with RA. Moreover, midodrine and rifaximin significantly reduced paracentesis frequency in comparison with the controls. Furthermore, the results of Rai et al.’s (2017) pilot study suggest that the combination therapy of midodrine and tolvaptan better controls ascites when compared with midodrine or tolvaptan alone.

The other adrenergic agent clonidine (α2-adrenergic agonist) presents similar effects to those of midodrine and may theoretically decrease the activity of the sympathetic nervous system and the release of norepinephrine. The co-administration of clonidine and diuretics induced an earlier diuretic response associated with fewer diuretic requirements and complications. Several trials revealed that clonidine combined with standard medical treatment effectively controlled ascites in liver cirrhosis (Lenaerts et al., 2006; Hutchinson & Davies, 2011; Singh et al., 2013). Although some published reports have confirmed the effectiveness of low, non-hypotensive doses of clonidine in adult cirrhotics with ascites, AASLD and EASLD do not currently recommend clonidine for RA management due to insufficient evidence (Runyon & AASLD Practice Guidelines Committee, 2009; European Association for the Study of the Liver, 2018). Further high-quality clinical trials that compare the efficacy of midodrine and clonidine in the treatment of RA are required.

Currently available medical treatments for RA are summarized in Table 1.

**Table 1:** Medical management of refractory ascites.

Treatment modalities	Recent studies and recommendations confirming benefits of the modality in RA management	Challenges and adverse effects	Impact on patient survival
**Pharmacotherapy**			
Diuretics	European Association for the Study of the Liver (2018)—only if kidney sodium excretion on diuretics exceeds 30 mmol/day, only when tolerated, otherwise discontinued	Dyselectrolytemia (hypo- or hyperkalemia, hyponatremia); muscle cramps, hyperglycemia, heart arrhythmia, mood changes, gynecomastia	None
Vasoconstrictors			
Midodrine	Solà et al. (2018), Rai et al. (2017), Guo et al. (2016), Runyon & AASLD (2013), Yang et al. (2010)	Limited effects, controls ascites without any renal or hepatic dysfunction	Undetermined, warrant further investigation
Terlipressin	Gow et al. (2016), Fimiani et al. (2011), Krag et al. (2007)	Limited data, reduction in the number of paracenteses required, not FDA approved in the USA and Japan	Undetermined, warrant further investigation
Clonidine	Singh et al. (2013), Yang et al. (2010)	Low, non-hypotensive doses improve ascites control in combination therapy with diuretics and midodrine	Undetermined, warrant further investigation
V2 receptor antagonists			
Tolvaptan	Kogiso et al. (2018), Rai et al. (2017), Yan et al. (2015)	High cost; hypernatremia, osmotic demyelination, myelinolysis, liver toxicity	Undetermined, warrant further investigation
**Interventional therapy**			
Repeated LVP (with i.e., albumin infusion eight g/L of ascitic fluid removed) first-line treatment for RA	European Association for the Study of the Liver (2018), Runyon & AASLD (2013), Bernardi et al. (2012), Titó et al. (1990), Ginès et al. (1988)	Post-paracentesis circulatory dysfunction	Improved
TIPS	European Association for the Study of the Liver (2018), Strunk & Marinova (2018), Bureau et al. (2017b), Gaba et al. (2015), Bai et al. (2014), Runyon & AASLD (2013)	HE, liver failure, shunt occlusion, infections, shunt migration, cardiovascular alterations/cardiac volume overload/, pulmonary hypertension	Improved
ALFApump	Solbach et al. (2018), Bureau et al. (2017a), Solà et al. (2017), Stirnimann et al. (2017)	Limited to experienced centers; a significant frequency of re-interventions for the device malfunction, plastic peritonitis related to the intra-abdominal catheter, acute kidney injury	Improved
CART	Hanafusa et al. (2017), Kozaki et al. (2016)	Expensive, elevation of body temperature, chills, decrease in blood pressure, allergic reactions	Improved
Liver transplantation—the only curative option for RA	European Association for the Study of the Liver (2018), Runyon & AASLD (2013)	Surgical procedure of relatively high risk, requires careful screening for eligible recipients, donor organs availability is its major limitation	Improved, significant long-term survival

**Note:**

ALFApump, automated low-flow ascites pump; CART, cell-free and concentrated ascites reinfusion therapy; FDA, the Food and Drug Administration; HE, hepatic encephalopathy; LT, liver transplantation; LVP, large-volume paracentesis, RA, refractory ascites.

## Hepatic hydrothorax

Pleural effusion that develops in a patient with the end-stage liver disease without cardiopulmonary comorbidities is called hepatic hydrothorax (HH) and is another serious complication of decompensated liver cirrhosis (Garbuzenko & Arefyev, 2017; Lv, Han & Fan, 2018). It affects approximately 5–10% of cirrhotics and is commonly seen on the right side (85% of cases), but sometimes also occurs on the left side (13% of cases) or bilaterally (2% of cases) (Lv, Han & Fan, 2018). Patients with HH frequently present with dyspnea and hypoxia early in the course of fluid accumulation. Although there is no an evidence-based consensus for the management of HH, according to the AASLD guidelines (Runyon & AASLD, 2013) the first-line therapy begins with medical treatment which includes a low sodium diet (4.6–6.9 g of salt per day) and diuretics administered in doses similar to those recommended for cirrhotic ascites. On the other hand, the EASL recommends diuretics and thoracentesis as the first-line management of HH (European Association for the Study of the Liver, 2010). Interventional therapy is indicated in symptomatic HH in cirrhotics who have failed medical treatment and have developed refractory HH. Therapeutic thoracentesis is the standard procedure for such patients. Although it is relatively safe, occasional complications may occur including pneumothorax, embolism, pleural empyema and chest wall infection (Lv, Han & Fan, 2018). Rarely, re-expansion pulmonary edema has been observed as a result of large-volume thoracentesis with subsequent increased microvascular permeability and inflammatory reactions (Garbuzenko & Arefyev, 2017). Therefore, it is recommended to stop fluid drainage from the pleural cavity when unpleasant sensations in the chest occur or when the pleural pressure at the end of exhalation decreases below −20 mmH_2_O. It is crucial to examine a pleural fluid sample to confirm the diagnosis and to rule out spontaneous bacterial empyema, as well as other etiology of pleural effusion (Al-Zoubi et al., 2016; Garbuzenko & Arefyev, 2017; Lv, Han & Fan, 2018).

In patients who need more than one therapeutic thoracentesis within 2 weeks, insertion of indwelling tunneled pleural catheter (ITPC) may be considered. Unfortunately, due to possible serious complications such as a massive protein, electrolyte and/or fluid loss, hemo- or pneumothorax, hepatorenal syndrome and secondary infection, chest tube placement may be used as a palliative measure and should be avoided in uncomplicated HH (Al-Zoubi et al., 2016; Garbuzenko & Arefyev, 2017; Lv, Han & Fan, 2018). Recently, ITPC has been proposed as an acceptable treatment alternative for HH refractory to conventional medical management. In this patient population, ITPCs provide symptomatic relief, but the morbidity and mortality still remain the major concerns with this treatment modality (Haas & Chen, 2017; Baig et al., 2018; Shojaee et al., 2019). Further studies are necessary to assess ITPC long-term safety and effectiveness in patients with HH.

Transjugular, intrahepatic portosystemic shunt remains the standard and first-line approach to patients with refractory HH (Lv, Han & Fan, 2018). By decompressing the portal system, TIPS has been confirmed to be effective not only for RA but also HH, especially if PTFE covered stents are used. Nevertheless, the procedure still serves as a bridge to liver transplantation due to a high likelihood of development of TIPS-related liver failure (Lv, Han & Fan, 2018).

The management of refractory HH may also include surgical interventions such as (1) chemical pleurodesis; (2) adjustment of diaphragmatic defects or fenestration with or without concomitant pleurodesis; (3) peritoneovenous or pleurovenous shunting; or (4) liver transplantation as the only definitive cure (Al-Zoubi et al., 2016; Garbuzenko & Arefyev, 2017; Lv, Han & Fan, 2018).

## Conclusions

Refractory ascites is a relatively common complication of liver cirrhosis. Due to RA’s unfavorable prognosis, it should be properly and quickly diagnosed based on the criteria that help to exclude cases of inadequately treated RA. Various treatment options are available for patients with RA, but currently, liver transplantation remains the best one. Vasoconstrictive agents provide a promising therapeutic choice for RA and may help in management while the patient awaits a liver transplant. However, rigorous evaluation of these agents in larger randomized trials is needed before recommendations for their widespread clinical use can be issued. For HH, the other serious complication of PH, there is no evidence-based effective treatment currently available. Therefore, orthotropic liver transplantation still remains the best treatment option for this subgroup of patients. For those who are not candidates, thoracentesis, TIPS, pleurodesis or selected surgical interventions are proposed to improve their quality of life.

## Additional Information and Declarations

### Data Availability

The following information was supplied regarding data availability: There is no raw data; this is a literature review.
